# Influence of pre-operative oral carbohydrate loading vs. standard fasting on tumor proliferation and clinical outcome in breast cancer patients ─ a randomized trial

**DOI:** 10.1186/s12885-019-6275-z

**Published:** 2019-11-08

**Authors:** Tone Hoel Lende, Marie Austdal, Anne Elin Varhaugvik, Ivar Skaland, Einar Gudlaugsson, Jan Terje Kvaløy, Lars A. Akslen, Håvard Søiland, Emiel A. M. Janssen, Jan P. A. Baak

**Affiliations:** 1 Department of Breast & Endocrine Surgery, Stavanger University Hospital, Helse Stavanger HF, P.O. Box 8100, N-4068 Stavanger, Norway; 2 Centre for Cancer Biomarkers CCBIO, Department of Clinical Medicine, Faculty of Medicine and Dentistry, University of Bergen, Jonas Lies vei 87, N-5012 Bergen, Norway; 3 Department of Research, Stavanger University Hospital, Helse Stavanger HF, P.O. Box 8100, N-4068 Stavanger, Norway; 4 Department of Pathology, Stavanger University Hospital, Helse Stavanger HF, P.O. Box 8100, N-4068 Stavanger, Norway; 5 Department of Pathology, Helse Møre og Romsdal HF, P.O. Box 1600, N-6026 Ålesund, Norway; 6 Department of Mathematics and Physics, University of Stavanger, P.O. Box 8600 Forus, N-4036 Stavanger, Norway; 7 Gades Institute, Laboratory Medicine Pathology, University of Bergen, Jonas Lies vei 87, N-5012 Bergen, Norway; 8 Department of Clinical Science, University of Bergen, Jonas Lies vei 87, N-5012 Bergen, Norway; 9 Risavegen 66, N-4056 Tananger, Norway; 10 Vierhuysen 6, 1921 SB Akersloot, Netherlands

**Keywords:** Breast cancer, Carbohydrate load, Proliferation, Insulin, Insulin c-peptide, IGF-1, IGFBP3, Tumor size, Relapse-free survival, Breast cancer-specific survival

## Abstract

**Background:**

Conflicting results have been reported on the influence of carbohydrates in breast cancer.

**Objective:**

To determine the influence of pre-operative per-oral carbohydrate load on proliferation in breast tumors.

**Design:**

Randomized controlled trial.

**Setting:**

University hospital with primary and secondary care functions in South-West Norway.

**Patients:**

Sixty-one patients with operable breast cancer from a population-based cohort.

**Intervention:**

Per-oral carbohydrate load (preOp™) 18 and 2–4 h before surgery (*n* = 26) or standard pre-operative fasting with free consumption of tap water (*n* = 35).

**Measurements:**

The primary outcome was post-operative tumor proliferation measured by the mitotic activity index (MAI). The secondary outcomes were changes in the levels of serum insulin, insulin-c-peptide, glucose, IGF-1, and IGFBP3; patients’ well-being, and clinical outcome over a median follow-up of 88 months (range 33–97 months).

**Results:**

In the estrogen receptor (ER) positive subgroup (*n* = 50), high proliferation (MAI ≥ 10) occurred more often in the carbohydrate group (CH) than in the fasting group (*p* = 0.038). The CH group was more frequently progesterone receptor (PR) negative (*p* = 0.014). The CH group had a significant increase in insulin (+ 24.31 mIE/L, 95% CI 15.34 mIE/L to 33.27 mIE/L) and insulin c-peptide (+ 1.39 nM, 95% CI 1.03 nM to 1.77 nM), but reduced IGFBP3 levels (− 0.26 nM; 95% CI − 0.46 nM to − 0.051 nM) compared to the fasting group. CH-intervention ER-positive patients had poorer relapse-free survival (73%) than the fasting group (100%; *p* = 0.012; HR = 9.3, 95% CI, 1.1 to 77.7). In the ER-positive patients, only tumor size (*p* = 0.021; HR = 6.07, 95% CI 1.31 to 28.03) and the CH/fasting subgrouping (*p* = 0.040; HR = 9.30, 95% CI 1.11 to 77.82) had independent prognostic value. The adverse clinical outcome of carbohydrate loading occurred only in T2 patients with relapse-free survival of 100% in the fasting group vs. 33% in the CH group (*p* = 0.015; HR = inf). The CH group reported less pain on days 5 and 6 than the control group (*p* <  0.001) but otherwise exhibited no factors related to well-being.

**Limitation:**

Only applicable to T2 tumors in patients with ER-positive breast cancer.

**Conclusions:**

Pre-operative carbohydrate load increases proliferation and PR-negativity in ER-positive patients and worsens clinical outcome in ER-positive T2 patients.

**Trial registration:**

CliniTrials.gov; NCT03886389. Retrospectively registered March 22, 2019.

## Background

Breast cancer is the most frequent malignancy among women [1], representing 12% of all new cancer cases and 25% of all cancers in women worldwide [2, 3]. In Norway, the incidence of breast cancer has doubled during the last 50 years. The lifetime risk for a Norwegian woman developing the disease is 10–12% [4]. A total of 570,000 women across the globe died of breast cancer in 2015, comprising 15% of cancer deaths among women [3]. Approximately 75% of all new breast cancers are luminal breast cancer subtypes, which express estrogen receptor (ER) and/or progesterone receptor (PR) [5]. The etiological factors of breast cancer comprise genetic, hormonal, environmental, and lifestyle-related elements [6]. Risk factors relating to the Western lifestyle, including lack of physical exercise, being overweight, certain hormonal and dietary factors, and diabetes mellitus type 2, have recently gained increased attention [2].

The effect of carbohydrate consumption on breast cancer incidence and outcome is probably mediated through three parallel routes. One route is through stimulation of the insulin/ insulin-like growth factor-1 (IGF-1) axis in epithelial breast cells, which comprises the insulin receptor (IR) [7] and IGF1 signaling pathways [8]. This results in crosstalk between cellular signaling systems and endocrine resistance in luminal breast cancers (i.e., ER-positive tumors) [9, 10]. Secondly, a substantial part of the insulin effect is mediated by paracrine signaling in the tumor micro-environment between adjacent adipocytes, fibroblasts, and the epithelial cancer cell. Signaling factors, such as ER, IR, IGF1-R, adiponectin, and leptin are involved [11]. Thirdly, alimentary glucose may affect cancer cells directly through the Warburg effect, which is an expedient switch that changes cellular energy metabolism from oxidative mitochondrial ATP production to cytoplasmic aerobic glycolysis [12]. This transition enables the proliferative cancer cells to produce both ATP for energy and ribose for DNA synthesis [13].

In human breast cancer patients, studies on the relationship between carbohydrate/glucose content in food and quantitative insulin characteristics are lacking. Insulin is a growth factor that increases proliferation and decreases apoptosis, and elevated levels of insulin are associated with different cancers, including breast cancer [14]. In breast cancer patients without diabetes, high insulin levels have been associated with a poor prognosis [15]. Insulin receptors have been detected on breast cancer cells [16], though there is conflicting evidence on whether insulin directly regulates cancer proliferation, and how fast such an effect will occur. Also, there is a research deficit on the influence of carbohydrates on clinical outcome or prognostic endpoint biomarkers such as proliferation. Generally, proliferation is measured by the mitotic activity index (MAI), phosphohistone-H3 (PPH3), and Ki-67 [17, 18]. The MAI and PPH3 estimate the number of cells in M phase (mitosis) and G_2_M phase, respectively, whereas Ki-67 detects all cells outside the G_0_phase. Notably, insulin influences cell cycle kinetics by more rapid transit through the G_1_ phase in ER-positive cells [7].

A meta-analysis has shown that, in patients undergoing abdominal surgery, administration of two per-oral carbohydrate loads administered 12–18 h, and again 2–4 h, before elective surgery reduces postoperative insulin resistance and leads to enhanced recovery after surgery (ERAS) [19]. During surgery, however, breast cancer cells are pushed into the circulation [20]. Moreover, due to the pre-operative oral carbohydrate load used in ERAS protocols, these cells may have a much better chance of survival and of forming viable metastatic foci [21, 22]. Pre-operative oral hyperglycemic loading may bring breast cancer cells into a favorable state to escape, divide, thrive, and survive during surgery, which may then lead to an inferior long-term prognosis for breast cancer patients [23]. Therefore, it is of great importance to gain more insight into the effects of pre-operative carbohydrate administration in breast cancer regarding insulin-related characteristics, proliferation, and clinical outcomes.

The cell cycle in breast cancer is fast enough to be influenced by the two pre-operative oral carbohydrate loads in ERAS protocols [24, 25]. We chose to use the MAI as our primary endpoint for proliferation. Our hypotheses were that an ERAS protocol comprising two oral carbohydrate loads will improve post-surgical recovery in breast cancer patients, the oral carbohydrate load will stimulate cellular signaling and increase proliferation as measured by the MAI, and pre-operative carbohydrate loading will lead to an adverse prognosis in breast cancer patients. A subgroup analysis of ER-positive patients was planned before the study was started.

Thus, the aim of this study was to investigate whether a pre-operative carbohydrate load according to a standard ERAS protocol influences tumor proliferation, postsurgical recovery, and/or clinical outcome.

## Methods

This population-based cohort of operable breast cancer patients was randomized into an intervention group receiving pre-operative per-oral carbohydrate loading or a control group comprising the standard fasting pre-operative protocol with unlimited access to drinking water. The investigation was an open-labeled study for the patient and breast surgeon. However, all researchers at the Department of Pathology and hormone laboratory were blinded to the intervention.

### Patients

**Fig. 1:**
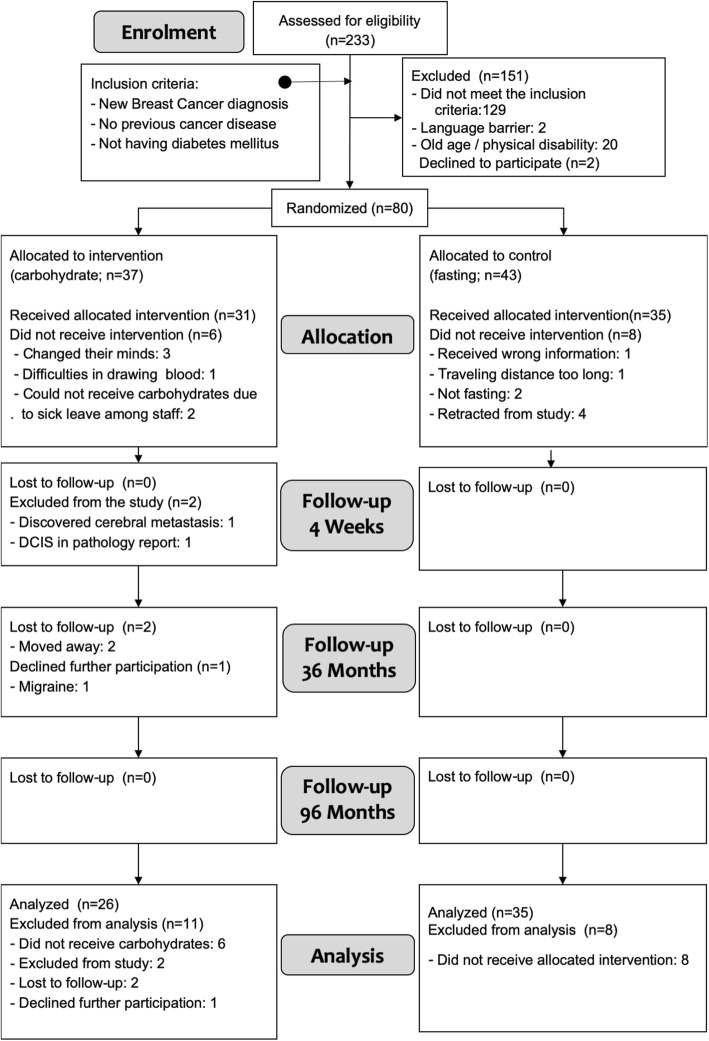
Study flow diagram

A total of 253 patients were assessed for eligibility between May 12, 2009, and June 23, 2010, in the catchment area of Stavanger University Hospital in South-West Norway. The exclusion criteria were clinical or radiological T3–4 tumors at clinical examination, overt systemic metastases, ductal carcinoma in situ (DCIS), micro-invasive cancer < 2 mm, or comorbidity, including diabetes mellitus type I and II, Cushing syndrome, previously diagnosed cancer, or being unable to co-operate in the study (e.g., dementia, other serious psychiatric illnesses, language barriers, or unwillingness to sign the informed consent papers). A total of 80 patients with unequivocal operable breast cancers (Stage I and II) diagnosed by fine needle aspiration cytology (FNAC) agreed to participate in the study and were randomized (Fig. 1). The last follow-up date was June 28, 2017. A larger proportion of dropouts in the intervention group for various random reasons created an imbalance in the numbers of patients between allocation groups (Fig. 1).

### Randomization and intervention

Randomization was performed after the patients provided written consent to participate in the study. The randomization procedure was organized as an in-house procedure with concealed envelopes generated and distributed in two boxes by the study nurse. The allocation sequence was performed by the trial administration committee. The sequence was balanced according to age by choosing between two boxes, one for age < 55 years (i.e., possible and certain premenopausal) and one for age ≥ 55 years (i.e., most probably postmenopausal), each with 1:1 block randomization regarding the carbohydrate (intervention) and fasting (control) groups in each box. The surgeon in the out-patient clinic enrolled consecutively operable breast cancer patients who agreed to participate in the trial.

### Intervention

Patients who were randomized to pre-operative carbohydrates drank 400 ml pre-Op™ (Nutricia, Netherlands) containing 12% carbohydrates, 2% glucose, and 10% polysaccharides the evening before (i.e., 18 h before surgery) and in the morning on the day of the operation (i.e., 2–4 h before surgery). Each patient was asked before surgery if they had been able to finish the carbohydrate drink or if they were fasting according to the randomization. The control group followed the standard fasting procedure with free intake of tap water.

### Blinding

The study was not blinded for the patients due to use of the carbohydrates and tap water by the participants. The information on the grouping was known only to THL, who was head of the clinical part of the trial, and this information was kept in a locked safe. Others involved in the study had no access to this information. Thus, the investigation was blinded for the laboratory personnel performing various assessments (MAI, PPH3, Ki67, histological grading, insulin, C-peptide etc.).

### Primary treatment

The primary surgery was performed according to the recommendations of the Norwegian Breast Cancer Group (NBCG) [4]. The surgery was either breast conserving treatment (BCT) or mastectomy, and sentinel node (SN) diagnostic or axillary lymph node clearance of level I and II. Adjuvant chemotherapy was also given based on the NBCG guidelines [4]. Notably, we found no differences between the two allocation groups regarding the type of primary treatment received (Table 1).

**Table 1:** Baseline characteristics of patients in the two study groups

Variable	Carbohydrate group (*n* = 26)	Missing data (Intervention group)	Fasting group (*n* = 35)	Missing data (Control group)	*P*
	*n* (%)		*n* (%)		
Age					
< 55	12 (46)	0	16 (46)	0	0.973
> 55	14 (54)	0	19 (54)	0	
BMI (kg/m^2^)	25.0 (3.9)	4	25.1 (3.0)	3	0.868
BMI < 25^a^	14 (64)	4	17 (53)	3	0.443
BMI ≥ 25	8 (36)		15 (47)		
BMI < 75 percentile^b^	18 (82)	4	23 (76)	3	
BMI ≥ 75 percentile	4 (18)		13 (24)		0.401
Menopausal status					
Premenopausal	4 (17)	1	7 (22)	1	0.627
Postmenopausal	20 (83)	1	25 (78)	2	
HRT - yes	8 (35)	3	10 (32)	4	0.937
HRT – no	14 (61)		19 (61)		
HRT- not relevant	1 (4)		2 (7)		
HRT use (years)	4.7 (4.3)	16	7.9 (5.8)	25	0.176
Tumor size (mm)	19.4	0	15.0	0	0.094
Tumor category					
T1	16 (62)	0	29 (83)	0	
T2	10 (38)	0	6 (17)	0	0.061
Histological Grade^c^					0.157
1	4 (15)	0	7 (20)	0	
2	10 (39)	0	20 (57)	0	
3	12 (46)	0	8 (23)	0	
pN negative	18 (69)	0	25 (71)	0	0.852
pN positive	8 (31)	0	10 (29)	0	
Number LNs removed	5.5	2	5.8	0	0.843
Number positive LNs	0.38	2	0.86	0	0.191
Estrogen receptor					
Positive (≥1%)	21 (81)	0	29 (83)	0	0.834
Negative (< 1%)	5 (19)	0	6 (17)	0	
Progesterone receptor					
Positive (≥ 10%)	13 (50)	0	28 (80)	0	0.014
Negative (< 10%)	13 (50)	0	7 (20)	0	
HER2					
Positive	3 (12)	0	1 (3)	0	0.176
Negative	23 (88)	0	34 (97)	0	
MAI (median, IQR)	7 (2–9)	1	5 (2–9)	0	0.647
MAI < 10	14 (56)	1	27 (77)	0	
MAI ≥ 10	11 (44)		8 (23)	0	0.083
Ki67 (mean, SD)	30.4 (28.2)	0	28.0 (26.5)	1	0.747
Ki67 < 15%	9 (35)	0	17 (50)	1	
Ki67 ≥ 15%	17 (65)	0	17 (50)	0	0.233
Ki67 < 30%	14 (54)	0	24 (71)	1	0.182
Ki67 ≥ 30%	12 (46)	0	10 (29)	0	
PPH3 (mean, SD)	20.2 (24.7)	0	20.5 (26.9)	0	0.966
PPH3 < 13	14 (54)	0	21 (60)	0	0.631
PPH3 ≥ 13	12 (46)	0	14 (40)	0	
TILs (mean %, SD)	4.7 (10.7)	0	4.3 (7.3)	1	0.137
TILs					
Positive (> 10%)	2 (8)	0	4 (11)	0	0.663
Negative (< 10%)	24 (92)	0	31 (89)	0	
Luminal type^d^					
Luminal A	16 (62)	0	23 (66)	0	
Luminal B	10 (38)	0	12 (34)	0	0.737
Glucose					
Admission^e^|	5.4 (1.1)	0	5.3 (0.6)	0	0.864
Pre-operative^f^	5.2 (1.8)	0	5.1 (0.6)	0	0.739
S-Insulin					
Admission^e^	9.4 (8.5)	0	9.1 (6.6)	0	0.886
Pre-operative^f^	33.7 (20.2)	0	9.1 (5.9)	0	< 0.0001
S-insulin-c-peptide					
Admission^e^	0.69 (0.32)	0	0.75 (0.32)	0	0.517
Pre-operative^f^	2.10 (1.05)	0	0.75 (0.27)	0	< 0.0001
Surgery					
BCT	15 (58)	0	23 (66)	0	
Mastectomy	11 (42)	0	12 (34)	0	0.523
Axillary staging					
SN	21 (81)	0	28 (80)	0	
ALND	5 (19)	0	7 (20)	0	0.940
Reoperation - 1					
-Breast	1 (20)	0	1 (50)	0	
-Axilla	4 (80)	0	1 (50)	0	0.427
Chemo therapy					
Yes	12 (46)	0	17 (47)	0	
No	14 (53)	0	18 (51)	0	0.852
Radiation therapy					
Yes	17 (68)	0	26 (74)	0	
No	8 (32)	1	9 (26)	0	0.594
Endocrine therapy					
Yes	17 (65)	0	22 (63)	0	
No	9 (35)	0	13 (37)	0	0.839
Smoking status		5		4	
-Never smoked	5 (24)		10 (32)		0.650
-Former smoker	9 (43)		14 (45)		
-Ongoing smoking	7 (33)		7 (23)		

Tumor size category analyzed as T1 vs. T2

^a^BMI-25 represents a dichotomized BMI <  25 or ≥ 25 on the BMI scale

^b^BMI-75p represents a dichotomized BMI with cut off < /≥ 75 percentile, i.e., </≥ 26.8 on the BMI scale

^c^Histological grading was performed according to the Nottingham algorithm

^d^Luminal A = ER+/HER2−/Ki67 < 15% and Luminal B = ER+/HER2−/Ki67 ≥ 15%

^e^Blood samples taken in the fasting state at the time patients were admitted in the hospital approx. 24–30 h before surgery

^f^Pre-operative blood samples taken 1–2 h before the surgical procedure commenced

*BMI* Body mass index, *HRT* Hormonal replacement therapy, *pT* Pathological tumor size in mm or category, *pN* Pathological lymph node status, *LN* Lymph node, *HER-2* Human epidermal growth factor receptor 2, *MAI* Mitotic activity index, *TILs* Tumor infiltrating leucocytes, *PPH3* Phosphorylated phospho-histone 3, *SN* Sentinel node, *ALND* Axillary lymph node dissection

### Safety issues

The patients were hospitalized for 1–2 days after surgery. Any complications, such as hemorrhage, infection, or others, were recorded on the Case Report Forms. No patients died or experienced any serious complications from the pre-operative treatment.

### Blood sampling for serum analysis

Five blood samples were obtained from the participants: 1) at the time of diagnosis, 2) at admission (the day before surgery), 3) pre-operatively before surgery, after the second pre-Op™ carbohydrate dose, 4) the day after surgery, and 5) 4 weeks post-surgery. Immediately after being drawn, the blood samples were put in ice water for transport to the in-house medical laboratory. The samples were spun and the serum frozen for transport to the Hormone Laboratory, Haukeland University Hospital, Bergen, Norway, where insulin, insulin c-peptide, IGF-1, and IGFBP-3 were measured by the IMMULITE 2000 two-site chemiluminescent immunometric assay (Siemens Medical Solutions Diagnostics).

### Histology

Tumor size was measured macroscopically in fresh specimens following excision. The tissues were cut into 0.5-cm slices. The axillary lymph nodes from sentinel node biopsy, or axillary fat from axillary dissection were examined macroscopically by a pathologist. All detectable lymph nodes (median 3 per patients, range 1–21) were prepared for histological examination. No lymph nodes were detected in two patients. For hematoxylin–eosin–saffron (HES) staining, the tissues were fixed in buffered 4% formaldehyde, embedded in paraffin, and sectioned (4 μm). The histological type and grade were assessed according to World Health Organization criteria (by two pathologists, EG and JPAB) [26].

### Immunohistochemistry

Immunohistochemistry (IHC) was performed to identify ER, PR, PPH3, Ki-67, and human epidermal growth factor receptor 2 (HER2) in whole sections. The antigen retrieval and IHC techniques were based on DAKO technology [27]. Formalin-fixed paraffin-embedded (FFPE) sections (4-μm thick) were serially sectioned after the preparation of HES sections and mounted onto siliconized slides (#S3002, DAKO, Glostrup, Denmark). A highly stabilized retrieval system (ImmunoPrep; Instrumec, Oslo, Norway) was used for antigen retrieval with the retrieval buffer (10 mM Tris/1 mM EDTA, pH 9.0). Sections were heated for 3 min at 110 °C, and then 10 min at 95 °C, before cooling to 20 °C. The following antibodies and dilutions were used: ER (clone SP1, Neomarkers/LabVision, Fremont, CA, USA), 1:400; PR (clone SP2, Neomarkers/LabVision), 1:1000; rabbit polyclonal anti-PPH3 (ser 10) (Upstate #06–570; Lake Placid, NY), 1:1500; and Ki-67 (clone MIB-1, DAKO, Glostrup, Denmark), 1:100. All antibodies were incubated for 30 min at 22 °C. Visualization was achieved using the EnVision™ FLEX detection system (DAKO, K8000). Sections were incubated with the peroxidase-blocking reagent (SM801) for 5 min, followed by the primary antibody for 30 min, EnVision™ FLEX/HRP Detection Reagent (SM802) for 20 min, EnVision™ FLEX DAB+ Chromogen (DM827)/EnVision™ FLEX Substrate Buffer (SM803) mix for 10 min, and EnVision™ FLEX Hematoxylin (K8008) for 5 min. Next, the slides were dehydrated, mounted, and stained using a Dako Autostainer Link 48 instrument and EnVision™ FLEX Wash Buffer (DM831). To assess HER2, the DAKO HercepTest™ was used according to the manufacturer’s protocol.

### Quantitative measures

MAI was assessed as the total number of mitotic figures in 10 consecutive fields of vision at 400× magnification (objective 40, specimen level field diameter 450 μm) in the most poorly differentiated periphery of the tumor, representing a total area of 1.59 mm^2^. Areas with necrosis or inflammation were avoided. This was performed as a routine diagnostic procedure, but controlled by EJ as described elsewhere [28]. We assessed the PPH3 index as described previously [29] and evaluated PPH3 expression using the fully automated VIS analysis system (Visiopharm, Hørsholm, Denmark) and previously described image processing principles [27]. The semi-automatic interactive computerized QPRODIT system (Leica, Cambridge) was used to measure the percentage of Ki-67-positive cells as described elsewhere [30]. A total of 250–350 fields of vision were systematically selected at random for each measurement. The Ki-67 percentage was defined as [(Ki-67 positive)/ (Ki-67 positive + Ki-67 negative)] × 100. ER-positivity was the presence of nuclear staining in > 1% of the cancer cells and ER-negative when < 1% of the cells were stained. For PR, positive was defined as nuclear staining present in > 10% of the cancer cells, borderline as 1–10% of the cancer cells exhibiting nuclear staining, and negative as < 1% of the epithelial breast cancer cells exhibiting nuclear staining. The DAKO Hercep-Test scoring protocol was used to score HER2, with 2+ and 3+ cases considered to be positive. Two of the authors (BH and EJ) scored all sections independently.

The relative number of stromal tumor-infiltrating lymphocytes (TILs) was assessed according to Salgado et al. [31]. HE-stained tissue sections were scored semi-quantitatively according to the presence or absence of stromal TILs. The degree of infiltration was scored from 0 to 100%, with positive TILs defined as ≥10%. Tumors were also classified as luminal A (ER+/HER2−/Ki67 < 15%) or luminal B (ER+/HER2−/Ki67 ≥ 15% or ER+/HER2+ regardless of Ki67) cancers according to the St. Gallen 2013 recommendations [32].

### Main outcome measures

The main primary outcome measure was the difference in proliferation (measured by MAI) in the primary tumor between the study groups. The secondary outcome measures were differences in insulin-related characteristics (i.e., insulin/c-peptide, IGF1, and IGFBP3) between the intervention and control groups. Patient-reported outcome measures (PROMs) on the following complaints and symptoms were also regarded as secondary outcomes: nausea, pain, mobilization, dizziness, insecurity, and bleeding. We applied an in-house questionnaire with which the patients were asked to score the six variables on a 4-step Likert scale (1 = ‘no’, 2 = ‘little’, 3 = ‘moderate’, and 4 = ‘very much’) on days 1, 2, 3, 4, 5, 6, and 7 after the operation.

For long-term outcome measures, we looked at relapse-free survival (RFS), defined as the time from surgery until the time the patient was diagnosed with a relapse in any location (i.e., locoregional, systemic, or contralateral). The time from surgery until death due to breast cancer was the breast cancer-specific survival (BCSS). The time from surgery until death from any cause constituted overall survival (OS). For both the primary and secondary outcomes, a subgroup analysis was planned for the ER-positive (luminal) breast cancer subtype.

### Statistical analysis

Power calculations were performed on the basis of the primary endpoint. We anticipated a 20% increase in MAI in the intervention group compared to the control group. Based on the mean value of MAI in patients belonging to the catchment area of Stavanger University Hospital [33, 34] and the reproducibility of the method to assess MAI, a total of 30 patients was needed in each study group (i.e., 60 patients) to achieve 80% power. We decided to randomize 80 patients to allow for a 10–15% drop-out rate.

As ER- positive breast cancer comprises approximately 75% of all breast cancer cases, there should be a reasonable number of patients to perform a subgroup analysis of luminal breast cancers. Statistical analyses were performed using SPSS statistical software v.22 (SPSS, Inc., Chicago, IL, USA). Differences in the clinical variables between the intervention groups were determined using T-tests, Fishers exact test, or chi-squared tests as appropriate. Kaplan-Meier survival curves were constructed, and the log-rank test was used to evaluate survival differences between groups. Cox proportional hazard analysis was used to test the relative importance of potential prognostic variables. In multivariable Cox regression, a backward stepwise model selection procedure was used, in which all covariates deemed clinically relevant were included in the initial model.

The proportion of patients reporting at least mild problems on each of the items on the PROM questionnaire each day for the first 7 postoperative days was analyzed using a mixed effects logistic regression model. Using this model, we tested for differences between the intervention and control groups. If a significant difference was found, a post-hoc analysis was performed using chi-squared tests for each of the days. We did not apply any correction for multiple testing due to the pilot and exploratory nature of the study. A two-tailed *P*-value of 0.05 was considered the threshold for significance.

### Manuscript reporting

We ensure that the manuscript reporting adheres to CONSORT guidelines for reporting clinical trials, including sticking to the CONSORT check list.

## Results

The various characteristics of the two allocation groups are shown in Table 1. Fifty patients had ER-positive tumors and 11 ER-negative tumors. Of the latter, 8 were HER2-negative (ER-, HER2-) and 4 were triple-negative (ER-, PR-, HER2-) based on IHC profiling. Notably, we found no differences in the distribution of the basic covariates between the carbohydrate-intervention group and the fasting group (Table 1).

### Proliferation markers

In the total study cohort, none of the continuous variables (MAI, Ki67, or PPH3) were different between the carbohydrate and fasting groups. However, when applying the robust and well-established prognostic threshold for MAI (< 10/≥10), among the ER-positive patients (*n* = 50) significantly more patients in the carbohydrate intervention (70%) had high proliferation (MAI ≥ 10) than in the fasting group (30%; *p* = 0.038; Table 2). The same trend was found when all tumors were considered (58% vs. 42%, carbohydrate vs. fasting; *p* = 0.083). In lymph node-negative luminal patients, the same correlation was stronger with a Kendall’s tau-b r = 0.488 (*p* = 0.017), Gamma r = 1.000 (*p* = 0.017), and Pearson chi-squared = 7.62 (*p* = 0.006; Fischer exact = 0.014 (two-sided); Table 3).

**Table 2:** Cross table MAI and allocation groups in ER+ patients

			Carbohydrate	Fasting	Total
	MAI < 10	Count	13	26	39
		%	65.0%	89.7%	79.6%
	MAI ≥ 10	Count	7	3	10
		%	35.0%	10.3%	21.4%
Total		Count	20	29	49
		%	100.0%	100.0%	100,0%

Pearson chi-squared: 4.430, df = 1, *p* = 0.035

Fischer exact: 0.041 (one-sided) and 0.068 (two-sided)

r (gamma) = 0.647 (*p* = 0.042)

r (Kendall’s tau-b) = 0.301 (*p* = 0.042)

**Table 3:** Cross table MAI and allocation groups in ER+ /LN negative patients

			Carbohydrate	Fasting	Total
	MAI < 10	Count	8	20	28
		%	66.7%	100.0%	87.5%
	MAI ≥ 10	Count	4	0	4
		%	33.3%	0.0%	12.5%
Total		Count	12	20	49
		%	100.0%	100.0%	100.0%

Pearson chi-squared: 7.619, df = 1, *p* = 0.006

Fischer exact: 0.014 (one-sided) and 0.014 (two-sided)

r (gamma) = 1.000 (*p* = 0.017)

r (Kendall’s tau-b) = 0.488 (*p* = 0.017)

### Progesterone receptor

Significantly more patients in the carbohydrate group had PR-negative tumors (50%) compared to the fasting group (20%; *p* = 0.014), independent of luminal A/B status.

### S*erum glucose and insulin responses*

**Fig. 2:**
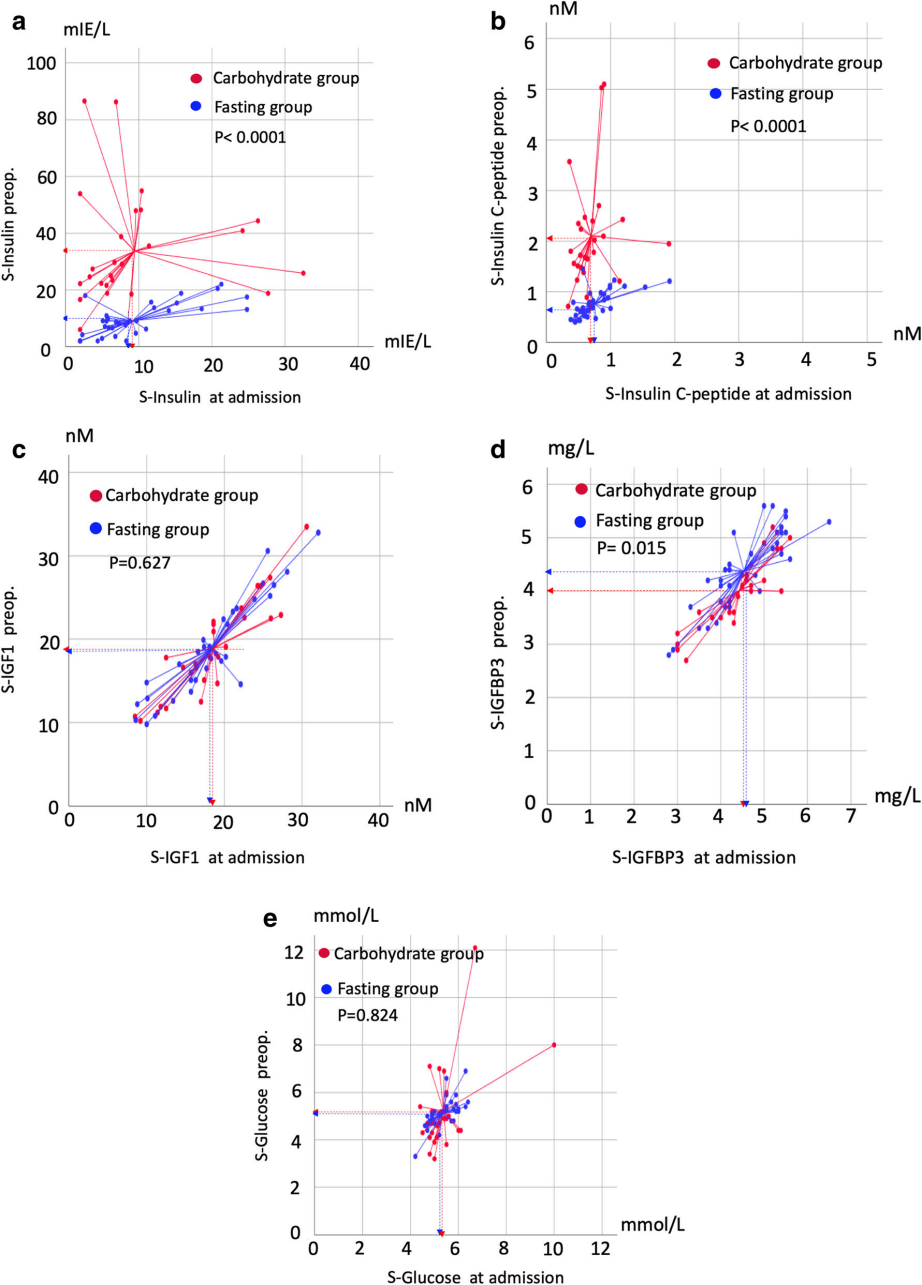
Scatterplot of the various insulin-related measures in serum in the two study groups. **a** S-insulin. **b** S-insulin c-peptide. **c** S-IGF. **d** S-IGFBP3. **e** S-glucose. The center of the centroid reference lines represents the mean value in each group (dotted lines). *P*-values were determined using t-tests. Units are given by the x-axis and y-axis. All values on the x-axis are at admission, and the y-axis values represent pre-operative measurements. Red, carbohydrate group; blue, fasting group; S, serum; Preop., pre-operatively; IGF, insulin-like growth factor; IGFBP3, IGF-binding protein 3

The response to pre-operative carbohydrate loading was assessed by the difference between the pre-operative serum values and the values obtained at admission (i.e., serum levels after carbohydrate loading minus fasting baseline values in both groups; Table 4). As expected, the intervention group had a significant increase in both S-insulin (+ 24.31 mIE/L, *p* <  0.0001, 95% CI 15.34 mIE/L to 33.27 mIE/L) and S-insulin c-peptide (+ 1.39 nM, *p* <  0.0001; 95% CI 0.21 nM to 0.97 nM). The upper quartile (Q_4_) border value of 2.40 nM was equal to the upper value of the normal range of insulin c-peptide (Table 4), indicating that 25% of the patients had c-peptide values compatible with insulin resistance. Regarding IGFBP3, a significant reduction of − 0.43 nM was measured after carbohydrate loading (*p* <  0.0001, 95% CI − 0.56 nM to − 0.27 nM) and − 0.26 nM compared to the control group (*p* = 0.015, 95% CI – 0.46 nM to – 0.051 nM). We found no changes in S-glucose or S-IGF-1 values within or between the two study groups (Table 4, Fig. 2a-f).

**Table 4:** Changes in glucose and insulin-related characteristics in the study groups

	Carbohydrate group (CH)				Fasting group (F)				Between groups		Normal range
	Admission values (A)	Pre-operative Values (Pop)	Difference within group (A-Pop)	P-diff within group	Admission values (A)	Pre-operative Values (Pop)	Difference within group (A-Pop)	P-diff within group	Difference between group CH vs. F	P-diff between groups	
GLUCOSE (mmol/L)											4.0 to 6.0
Median	5.05	4.70			5.30	5.10					
Mean	5.37	5.22	– 0.15	0.625	5.34	5.11	– 0.23	0.009	+ 0.072	0.824	
IQR	4.88 to 5.05	4.25 to 5.50			4.80 to5.80	4.70 to 5.40					
Range	4.40 to 10.00	3.2 to 12.1			4.20 to 6.40	3.30 to 6.90					
95% CI	4.90 to 5.80	4.48 to 5.96	– 0.79 to 0.49		5.15 to 5.53	4.89 to 5.33	−0.39 to −0.061		−0.59 to 0.73		
INSULIN (mIE/L))											< 29.1
Median	6.80	26.65			6.90	8.60					
Mean	9.43	33.68	+ 24.25	< 0.0001	9.14	9.09	– 0.58	0.940	+ 24.31	< 0.0001	
IQR	3.60 to 10.33	20.90 to 45.28			5.00 to 12.1	3.30 to 8.60					
Range	2.00 to 32.50	6.00 to 86.60			2.00 to 24.80	2.00 to 22.00					
95% CI	6.00 to 12.90	25.52 to 41.85	15.39 to 33.11		6.88 to 11.04	7.06 to 11.12	−1.58 to 1.47		15.34 to 33.27		
C-PEPTIDE (nM)											< 2.4
Median	0.61				0.66	0.68					
Mean	0.70	2.10	+ 1.40	< 0.0001	0.75	0.76	+ 0.004	0.926	+ 1.39	< 0.0001	
IQR	0.50 to 0.83	1.50 to 2.41			0.53 to 0.66	0.53 to 0.68					
Range	0.34 to 1.91	0.71 to 5.10			0.38 to 1.92	0.40 to 1.45					
95% CI	0.57 to 0.83	1.67 to 2.53	0.98 to 1.83		0.64 to 0.86	0.66 to 0.85	– 0.083 to 0.091		1.03 to 1.77		
IGF-1 (nM)											5 to 28
Median	18.60	18.45			18.10	17.90					
Mean	18.67	18.98	+ 0.31	0.541	18.30	18.88	+ 0.58	0.145	+ 0.62	0.672	
IQR	14.2 to 23.0	14.15 to 18.45			15.70 to 21.60	18.80 to 23.30					
Range	8.5 to 30.6	10.20 to 33.50			8.60 to 32.80	9.80 to 32.80					
95% CI	16.36 to 20.98	16.59 to 21.37	−0.72 to 1.35		16.34 to 20.25	16.89 to 20.86	– 0.21 to 1.36		−1.51 to 0.98		
IGFBP-3 (mg/L)											2.9 to 5.1
Median	4.55	4.05			4.20	4.40					
Mean	4.43	4.02	−0.42	< 0.0001	4.53	4.37	– 0.16	0.042	– 0.26	0.015	
IQR	3.95 to 5.05	3.50 to 4.58			4.00 to 5.30	3.80					
Range	3.00 to 5.60	2.70 to 5.20			2.80 to 6.50	2.80 to 5.60					
95% CI	4.11 to 4.75	3.74 to 4.30	−0.56 to −0.27		4.24 to 4.83	4.11 to 4.64	−0.31 to − 0.0061		−0.46 to − 0.051

### Quality of life data

In the carbohydrate intervention group, fewer patients reported mild and moderate pain during the first 7 postoperative days than in the fasting group (*p* <  0.001), which in post-hoc analysis was significant on postoperative day 5 (28% vs. 47%; *p* = 0.038) and day 6 (28% vs. 50%; *p* <  0.001). Otherwise, there were no significant differences between the two groups regarding the other items from the PROM questionnaire (nausea, mobilization, dizziness, insecurity, and bleeding) (data not shown).

### Long-term clinical outcome

The median follow-up for RFS was 88 months (range 33 to 97 months) and for BCSS 88 months (range 45 to 97 months). Eight patients experienced a relapse: one loco-regional, six systemic, and one contralateral. Five of these patients died of breast cancer.

### Relapse-free survival

**Fig. 3:**
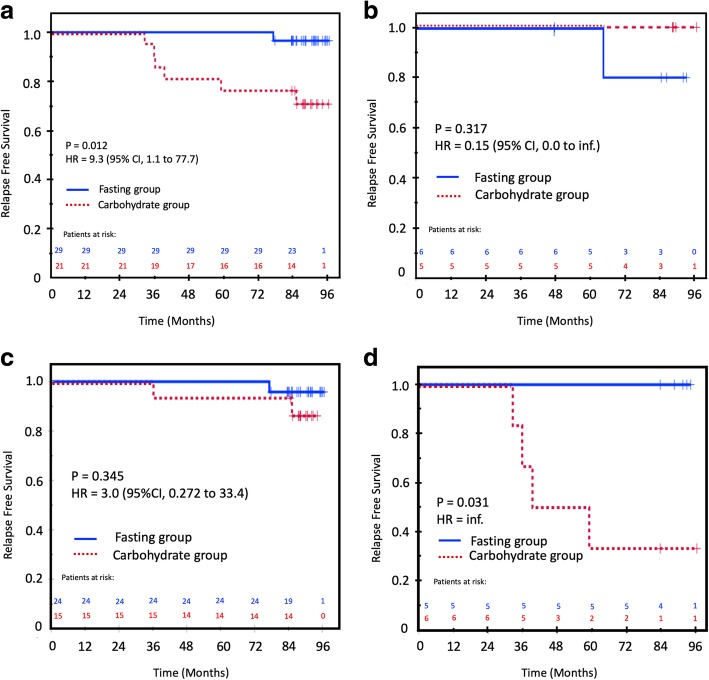
Relapse-free survival (RFS) in the carbohydrate and fasting groups. **a** In all ER-positive patients. **b** In all ER-negative patients. **c** In ER-positive, T1 patients. **d** In ER-positive, T2 patients. Fasting group, blue solid line; carbohydrate group, red dotted line. Patients at risk are above the X-axis with the same color coding as the treatment groups. Censored patients are marked with a + sign on the survival curves

Randomization to intervention with pre-operative carbohydrates had a weak and borderline influence on RFS when analyzed in the whole study cohort (Table 5). However, in the ER-positive patients who received carbohydrates pre-operatively, a reduced RFS of 71% compared to 97% in the control group (*p* = 0.012, HR = 9.3, 95% CI 1.1 to 77.7; Table 5 and Fig. 3a) was observed. The covariates tumor diameter between 2 and 5 cm (T2) and the proliferation marker Ki67 (both ≥15% and ≥ 30%) had a significant negative influence on RFS in both the whole cohort and in the ER-positive cohort (Table 5). In the ER-negative subgroup, there was no influence of the carbohydrate/fasting grouping on RFS (Fig. 3b). The following co-variates were deemed clinically relevant: tumor size, nodal status, histological grade, PR and HER2 status, Ki67–15%, Ki67–30%, PPH3–13, MAI-10, TILs, luminal A/B status, carbohydrate/fasting grouping, chemotherapy, radiotherapy and endocrine therapy, BMI-75p, BMI-25, and smoking status. In the multivariable analysis, tumor size (T1/T2; *p* = 0.021, HR = 6.07, 95% CI = 1.31 to 28.03) and carbohydrate/fasting grouping (*p* = 0.040; HR = 9.30, 95% CI 1.11 to 77.82) were the only two variables left in the final Cox model. As T2 tumors were more frequent in the intervention group, we performed a Kaplan Meier analysis of the influence of the carbohydrate intervention on RFS stratified for T1 vs. T2. This analysis showed that the unfavorable prognostic effect of carbohydrate loading was not present in the T1 (≤ 2 cm) patients, but was strongly prognostic in the T2 patients (Fig. 3c and d). In the T2 group, the carbohydrate-loaded and fasting patients had an RFS of 33 and 100%, respectively (*p* = 0.031; HR = inf). In the T2 subgroup, there was a significantly higher mean serum level of pre-operative insulin c-peptide among patients who experienced a relapse versus those who were relapse-free (2.02 nM vs. 0.838 nM, *p* = 0.025). Notably, there was an even distribution of luminal A and luminal B tumors among the patients with T2 tumors who experienced a relapse versus those who did not (*p* = 0.47).

**Table 5:** Univariable analysis of relapse-free survival

	Whole cohort (*n* = 61)				ER positive patients (*n* = 50)			
Characteristics	Event/at risk (% survival)	Log rank P	HR	95% CI	Event/at risk (% survival)	Log rank P	HR	95% CI
Pre-operative randomization								
Fasting	2/35 (94)	0.049	1		1/29 (97)		1	
Carbohydrates	6/26 (77)		4.4	0.9 to 21.7	6/21 (71)	0.012	9.3	1.1 to 77.7
Nodal status								
N0	3/43 (93)		1		3/33 (91)		1	
N+	5/18 (13)	0.03	9.8	1.10 to 88.1	4/17 (77)	0.16	2.8	0.63 to 12.6
Tumor size								
T1	3/45 (93)		1		3/39 (92)		1	
T2	5/16 (69)	0.009	5.5	1.3 to 23.2	4/11 (64)	0.008	6.0	1.3 to 27.0
Nottingham grade^b^		0.33				0.31		
Grade 1	0/11 (100)		1		0/11 (100)		1	
Grade 2	5/30 (83)	–	Inf.	Inf.	5/30 (83)		Inf.	Inf.
Grade 3	3/20 (85)	–	inf.	Inf.	2/9 (78)		inf.	Inf.
Estrogen receptor					–	–	–	–
Positive (≥ 1%)	7/50 (86)		1		–	–	–	–
Negative (< 1%)	1/11 (91)	0.67	1.6	0.2 to 12.7	–	–	–	–
Progesterone receptor								
Positive (≥10%)	4/41 (37)		1		3/37 (92)		1	
Negative (< 10%)	4/20 (80)	0.27	2.1	0.5 to 8.6	4/13 (69)	0.048	4.0	0.90 to 18.1
HER2								
Negative (0 to 1+)	7/57 (88)		1		6/49 (88)		1	
Positive (2+ to 3+)	1/4 (75)	0.46	2.1	0.3 to 17.5	1/1 (0)	0.005	11.7	1.3 to 105.1
MAI								
< 10	5/41 (88)		1		4/39 (90)		1	
≥ 10	3/19 (66)	0.66	1.4	0.3 to 5.8	3/10 (70)	0.09	3.4	0.8 to15.2
MAI								
< 3	2/16 (88)		1		2/16 (88)		1	
≥ 3	6/44 (86)	0.89	1.1	0.2 to 5.5	5/33 (85)	0.80	1.2	0.2 to 6.4
PPH3								
< 13	3/35 (91)		1		3/35 (91)		1	
≥ 13	5/26 (81)	0.26	2.2	0.5 to 9.4	4/15 (73)	0.12	3.1	0.7 to 14.0
Ki67								
< 15	0/26 (100)				0/25 (100)		1	
≥ 15	8/34 (77)	0.008	–	–	7/24 (71)	0.003	^a^	^a^
Ki67								
< 30	3/38 (92)		1		3/37 (92)		1	
≥ 30	5/22 (77)	0.093	3.2	0.8 to 13.4	4/12 (67)	0.023	4.8	1.1 to 21.8
TILs								
Negative (< 10%)	2/13 (85)				7/55 (87)		1	
Positive (≥10%)	6/48 (88)	0.77	1.4	0.2 to 3.9	1/6 (83)	0.75	2.2	0.24
Luminal status^c^								
Luminal A	3/39 (92)				2/28 (93)			
Luminal B	5/22 (77)	0.091	3.2	0.77 to 13.5	5/22 (77)	0.11	3.5	0.68 to 18.1
Chemotherapy								
Yes	6/29 (79)		1		5/20 (75)		1	
No	2/32 (94)	0.096	0.28	0.06 to 1.4	2/30 (93)	0.069	0.25	0.05 to 1.3
Radiotherapy								
Yes	6/43 (86)		1		5/38 (87)		1	
No	2/17 (88)	0.90	0.91	0.18 to 4.5	2/12 (83)	0.72	1.4	0.26 to 7.0
Endocrine Therapy								
Yes	7/39 (82)		1		6/36 (83)		1	
No	1/22 (96)	0.15	0.24	0.03 to 2.0	1/14 (93)	0.38	0.40	0.05 to 3.3
BMI-25^d^								
< 25	3/31 (90)		1		3/26 (89)		1	
≥ 25	4/23 (83)	0.40	1.9	0.42 to 8.4	3/20 (85)	0.70	1.4	0.28 to 6.8
BMI-75p^e^								
< 75p	4/41 (90)		1		4/36 (89)		1	
≥ 75p	3/13 (77)	0.201	2.57	0.57 to 11.5	2/10 (80)	0.417	1.99	0.36 to 10.9
Smoking								
-Never smoked	4/15 (73)		1		3/12 (87)		1	
-Former smoker	1/23 (96)		0.22	0.025 to 2.00	1/20 (95)		0.26	0.027 to 2.5
-Ongoing smoking	1/14 (93)	0.065	0.14	0.015 to 1.22	1/12 (92)	0.15	0.17	0.017 to 1.6

*BMI* Body mass index, *HRT* Hormonal replacement therapy, *T* Tumor size in mm or category, *N* Pathological lymph node status, *LN* Lymph node, *N0* Node negative, *N+* Node positive (assessed by pathologists), *HER-2* Human epidermal growth factor receptor 2, *MAI* Mitotic activity index, *TILs* Tumor infiltrating leucocytes, *PPH3* Phosphorylated phospho-histone 3

^a^HR (95% CI) was not computed, as the equation did not converge, and no events occurred in one or more categories

^b^Histological grading was performed according to the Nottingham algorithm

^c^Luminal A = ER+/HER2−/Ki67 < 15% and Luminal B = ER+/HER2−/Ki67 ≥ 15% or ER+/HER2 +

^d^BMI-25 represents a dichotomized BMI < 25 or ≥ 25 on the BMI scale

^e^BMI-75p represents a dichotomized BMI with cut off < /≥ 75 percentile, i.e., </≥ 26.8 on the BMI scale

### Breast cancer-specific survival

**Fig. 4:**
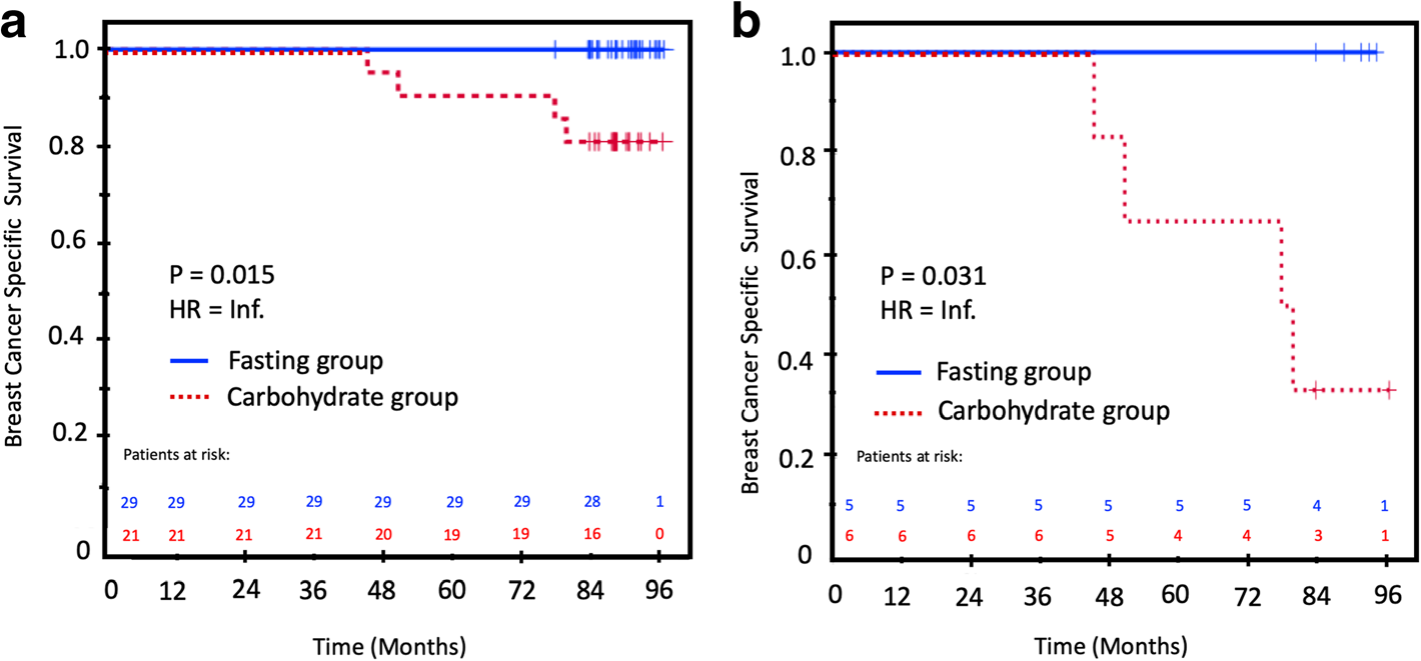
Breast cancer-specific survival (BCSS) in the intervention and control groups. **a** In all ER-positive patients. **b** In ER-positive, T2 patients Fasting group, blue solid line; carbohydrate group, red dotted line. Patients at risk are above the X-axis with the same color coding as the treatment groups. Censored patients are marked with a + sign on the survival curves

In the unadjusted analysis of BCSS, intervention with carbohydrates resulted in a significantly inferior BCSS in ER-positive patients compared to the control group (Table 6; Fig. 4a). In ER-positive T2 tumors, the carbohydrate intervention group had the worst BCSS (30%), compared to 100% in the control fasting group (p = 0.031, HR = infinite, due to zero relapses in one of the two groups; Fig. 4b). In addition, tumor size, nodal status, and Ki67–30% provided significant prognostic information in the unadjusted analysis (Table 6). In the multivariable analysis, only Ki67–30 remained in the final model. In general, the small number of patients and endpoints hampered a robust multivariable analysis.

**Table 6:** Univariable analysis of breast cancer-specific survival

Variable	Whole study cohort (*n* = 61)				ER-positive patients (*n* = 50)			
	Event/at risk (% survival)	Log rank *P*	HR^a^	95% CI	Event/at risk (% survival)	Log rank *P*	HR^a^	95% CI
Pre-operative randomization								
Fasting	1/35 (97)		1		0/29 (100)		1	
Carbohydrates	4/26 (85)	0.086	4.4	0.88 to 21.7	4/21 (81)	0.015	^a^	^a^
Nodal status								
N0	1/43 (98)				1/33 (82)		1	
N+	4/18 (78)	0.012	4.4	1.05 to 18.5	3/17 (82)	0.080	2.80	0.63 to 12.6
Tumor size			–	–				
T1	0/45 (100)		1		0/40 (100)		1	
T2	5/16 (69)	< 0.0001	5.5	1.32 to 23.1	4/10 (60)	< 0.0001	^a^	^a^
Nottingham grade ^b^		0.556				0.352		
Grade 1	0/11 (100)		1		0/11 (100)		1	
Grade 2	3/30 (90)		^a^	^a^	3/30 (90)		^a^	^a^
Grade 3	2/20 (90)		^a^	^a^	1/9 (89)		^a^	^a^
Estrogen receptor					–	–	–	–
Positive (≥ 1%)	4/50 (92)		1		–	–	–	–
Negative (< 1%)	1/11 (91)	0.852	0.64	0.079 to 5.21	–	–	–	–
Progesterone receptor								
Positive (≥10%)	4/41 (90)		1		3/37 (92)		1	
Negative (< 10%)	1/20 (95)	0.543	0.51	0.057 to 4.59	1/13 (92)	0.94	0.93	0.1 to 8.9
HER2								
Negative (0 to 1+)	4/57 (93)		1		3/49 (94)		1	
Positive (2+ to 3+)	1/4 (75)	0.248	3.37	0.38 to 30.2	1/1 (0)	0.001	11.7	1.31 to 105.1
MAI								
< 10	3/41 (93)		1		3/39 (92)		1	
≥ 10	2/19 (90)	0.645	1.5	0.25 to 9.1	2/10 (80)	0.23	4.1	0.6 to 29.3
MAI								
< 3	1/16 (94)		1		1/16 (88)		1	
≥ 3	4/44 (91)	0.735	1.46	0.16 to 13.0	3/33 (85)	0.76	1.4	0.15 to 13.8
PPH3								
< 13	2/35 (94)		1		2/35 (94)		1	
> 13	3/26 (89)	0.426	2.0	0.34 to 12.2	2/15 (87)	0.40	2.3	0.32 to 16.1
Ki67								
< 15	0/26 (100)		1		0/25 (100)		1	
≥ 15	5/34 (82)	0.040	–	–	4/24 (83)	0.014	^a^	^a^
Ki67								
< 30	1/38 (97)		1		1/37 (95)		1	
≥ 30	4/22 (82)	0.033	7.5	0.84 to 67.5	3/12 (75)	0.023	9.9	1.03 to 95.3
TILs								
Negative	4/55 (93)		1		3/45 (93)		1	
Positive	1/6 (83)	0.479	2.16	0.24 to 19.4	1/4 (75)	0.24	3.6	0.37 to 34.6
Luminal status^c^								
Luminal A	3/39 (92)		1		2/28 (93)		1	
Luminal B	2/22 (91)	0.847	1.2	0.20 to 7.41	2/22 (91)	0.777	1.33	0.19 to 9.42
Chemo therapy								
Yes	5/39 (87)		1		3/20 (85)		1	
No	0/22 (100)	0.089	0.22	0.024 to 1.95	1/30 (97)	0.15	0.22	0.023 to 2.10
Radiation therapy								
Yes	3/43 (93)		1		2/38 (95)		1	
No	2/17 (88)	0.499	1.84	0.33 to 11.0	2/12 (83)	0.19	3.9	0.48 to 24.1
Endocrine therapy								
Yes	5/39 (87)		1		4/36 (89)		1	
No	0/22 (100)	0.089	0.024	0 to 46.4	0/14 (100)	0.20	0.03	0 to 262
BMI-25^d^								
< 25	1/31 (97)		1		1/26 (96)		1	
≥ 25	3/23 (87)	0.177	4.19	0.44 to 40.3	2/20 (90)	0.398	2.70	0.25 to 29.8
BMI-75p^e^								
< 75p	2/41 (95)		1		2/36 (94)		1	
≥ 75p	2/13 (85)	0.218	3.20	0.45 to 22.8	1/10 (90)	0.622	1.81	0.16 to 20.0
Smoking								
-Never smoked	3/15 (80)		1		2/12 (83)		1	
-Former smoker	0/23 (100)		0.003	Inf.	0/20 (100)		0.003	Inf.
-Ongoing smoking	0/14 (100)	0.020	0.003	Inf	0/12 (100)	0.052	0.003	Inf.

*BMI* Body mass index, *HRT* Hormonal replacement therapy, *T* Pathological tumor size in mm or category, *LN* Lymph node, *N0* Node negative, *N+* Node positive (assessed by pathologists), *HER-2* Human epidermal growth factor receptor 2, *MAI* Mitotic activity index, *TILs* Tumor infiltrating leucocytes, *PPH3* Phosphorylated phospho-histone 3

^a^HR (95% CI) was not computed, as the equation did not converge and no events occurred in one or more categories

^b^Histological grading was performed according to the Nottingham algorithm

^c^Luminal A = ER+/HER2−/Ki67 < 15% and Luminal B = ER+/HER2−/Ki67 ≥ 15%

^d^BMI-25 represents a dichotomized BMI < 25 or ≥ 25 on the BMI scale

^e^BMI-75p represents a dichotomized BMI with cut off < /≥ 75 percentile, i.e., </≥ 26.8 on the BMI scale

### Overall survival

**Fig. 5:**
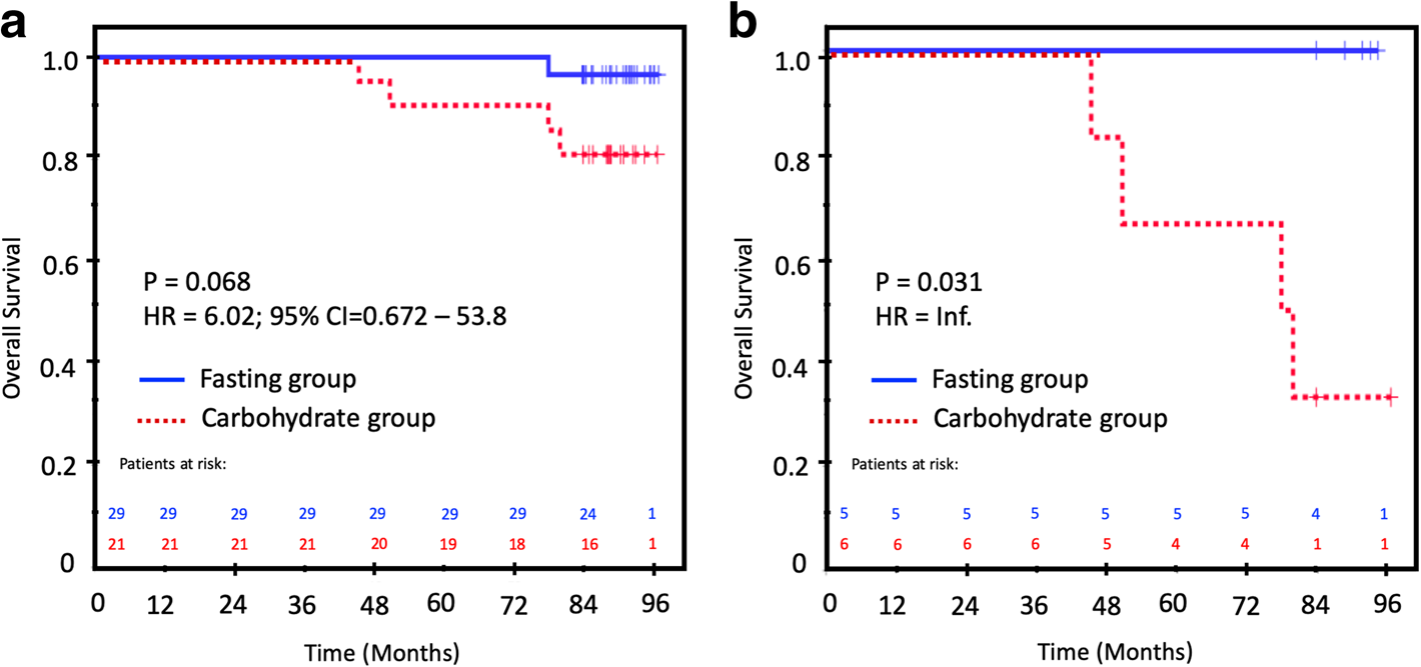
Overall survival (OS) in the intervention and control groups. **a** In all ER-positive patients. **b** In ER-positive, T2 patients. Fasting group, blue solid line; carbohydrate group, red dotted line. Patients at risk are above the X-axis with the same color coding as the treatment groups. Censored patients are marked with a + sign on the survival curves

The univariate analysis of OS in ER+ patients showed only a borderline significance of OS for the carbohydrate group (81%) compared to the fasting group (99%; *p* = 0.068; HR = 6.02; 95% CI 0.672–53.8; Fig. 5a). Only tumor size remained as an explanatory factor in the final Cox model (HR = 17.1; 95% CI 17.1–153). In the ER+/T2 patients, the corresponding OS was 33% vs. 100%, respectively (*p* = 0.031; HR = inf; Fig. 5b). In the Cox model, carbohydrate/fasting status was entered in the last step, but the model was considered too unstable for a reliable report.

### Adverse events

No adverse events were recorded in either of the two study arms. No signs of pathologically elevated fasting blood sugar levels (i.e., > 6 mmol/L) was noted. Furthermore, in the carbohydrate arm, no signs of occult diabetes mellitus were seen (i.e., blood sugar levels > 10 mmol/L) after carbohydrate loading.

## Discussion

Glucose has been correlated with cancer for nearly a century. Warburg (1925) was the first to describe the phenomenon that cancer cells have a much stronger tendency to take up glucose [35], for which (amongst other findings) he received the Nobel prize in 1932 [36]. However, to the best of our knowledge, the current study is the first prospective randomized trial to evaluate the effects of pre-operative carbohydrate loading on tumor proliferation and outcome (short-term vs. long-term) in operable breast cancer patients. In patients with ER-positive tumors (i.e., luminal tumors), significantly more patients with MAI ≥ 10 were observed in the carbohydrate group than the fasting group. Luminal cancers have, on average, a lower proliferation rate than ER-negative and triple-negative cancers [37]. As such, the proliferation-increasing effect of carbohydrate loading in luminal cancers understandably leads to a higher percentage increase in patients crossing the prognostically essential MAI-10 threshold. Most ER−/triple-negative breast cancer patients already have an MAI greatly exceeding 10. Therefore, carbohydrate loading will probably not increase proliferation in a clinically significant manner, as they have an a priori high risk of distant metastases [38]. In addition, the luminal A patients exposed to excess carbohydrates may turn into luminal B tumors, thereby statistically increasing their risk for recurrence. This is in agreement with luminal breast cancers responding directly to an increase in circulating insulin through altered transmembrane IRs [39]. Thus, in the present study, the observation of an increase in insulin/c-peptide in the intervention group could explain the increased MAI and Ki67 in the ER-positive group. Similarly, as triple-negative cancers better utilize the IGFBP3 pathway in EGF1-signaling [40], the observed reduction in IGFBP3 after carbohydrate loading may account for the lack of response to proliferation in the ER-negative group. This could suggest that the differential responses to the insulin/IGF1 axis between luminal and triple-negative cancers [41] explain our observed differences in response to per-oral carbohydrate loading and mitotic activity between the ER-positive and ER-negative groups.

The observed inferior RFS for ER-positive T2 tumors suggests that larger tumor size may influence the extent to which cancer cells activate all necessary features to promote the epithelial-mesenchymal transition (EMT) [42] and seed out micro-metastases. These processes turn into clinically overt relapses after some years [43]. This is in line with other research that has found a positive correlation between tumor size and relapse [44], and between tumor size and the development of endocrine resistance [45]. A crucial question is to what extent the pre-operative carbohydrate load to patients in the present study promoted the EMT process in the T2-T3 tumors and created more micro-metastases [46, 47]. Importantly, increased signaling through the insulin/IGF axis is known to promote both the EMT process [48] and chemotaxis [49], which increases the risk for minimal residual disease to occur. Furthermore, the pre-operative carbohydrates may have been administered in a critical window of the cancer’s life cycle. The number of liberated circulating tumor cells (CTCs) from the primary tumor sharply increases during surgery [50]. Thus, the administered carbohydrates may have given the CTCs systemic biological support with a triple survival benefit through the Warburg effect [12], the insulin/IGF-1 axis [51], and paracrine signaling with distant adipocytes [11]. Furthermore, increased IR/IGF-signaling promotes protein synthesis in the same way the PR pathway does. Consequently, the upregulation of IR/IGF-signaling will suppress the transcription of PR in the cell [52], which is considered to be part of the endocrine switch. Moreover, dietary carbohydrates may down-regulate the gene expression of PR through epigenetic mechanisms [53]. These mechanisms support our finding of less PR-positivity in the carbohydrate arm. Taken together, these components of the endocrine switch make CTCs more resilient to the adjuvant endocrine treatment following surgery [9, 54]. The present study seems to support the novel principle of manipulating the perioperative nutrient status for adjuvant treatment purposes. Recently, the complete opposite situation with a postoperative low carbohydrate/ketogenic diet was advocated in pancreatobiliary cancer surgery as an option for adjuvant anti-cancer therapy [55].

As the distribution of larger tumor sizes was skewed to the carbohydrate group, there may be another explanation than statistical chance. As the carbohydrates affected proliferation, they may have also affected the growth of tumor cells in the tumor periphery, where the MAI is measured. This may have resulted in more blurry demarcations of the tumor, which then interferes with the accuracy of measuring the tumor size. Thus, the increased tumor size in the carbohydrate group may have a biological basis.

The inferior prognosis of patients who received the carbohydrate load and had T2 tumors requires some reflection. Patients with higher levels of insulin c-peptide may be more responsive not only to the carbohydrate loading they received in the present study, but also to carbohydrates in every meal they consume during the period in which they receive adjuvant therapies and thereafter. These patients may have a subclinical insulin-resistant state, which is known to be a risk factor for relapse from breast cancer in non-diabetic women [56]. Therefore, tumor size combined with insulin c-peptide status may predict an increased effect of adjuvant metformin or other insulin-lowering drugs in the treatment of breast cancer patients. Metformin attenuates the systemic biological effect of IR/IGF on tumor-promoting signaling by improving insulin sensitivity and suppressing liver glucose output, which leads to reduced levels of systemic circulating insulin [14]. This further mitigates paracrine signaling, overcoming endocrine resistance [51, 57] and improving prognosis in breast cancer [58–61]. The present study supports the hypothesis that adjuvant metformin or other insulin-lowering therapeutic interactions may have their greatest effect in breast cancer patients with ER-positive T2 tumors. In addition, the greatly increased glucose consumption by cancer cells as measured by positron emission tomography (PET) with the tracer ^18^F-deoxy-glucose (FDG) [62] identifies patients with an inferior clinical outcome [63]. This may also serve as a promising proxy for insulin/metformin responders.

The effect of carbohydrate loading on well-being had a very limited clinical subjective effect in the present study (i.e., only reduced pain on the 5th and 6th day after surgery). Notably, no difference in mobilization or hospitalization was found. This is probably due to the short duration of the operation and the extraperitoneal nature of the surgical procedure in breast cancer patients. The health authorities in Norway recently introduced new national guidelines for a more standardized trajectory in breast cancer, without preoperative carbohydrate loading included [64]. Day-care surgery comprising anesthesiology medication with a short half-life, leading to fewer side effects for the patients and an optimized pain relief regimen, has been introduced since this trial was performed. Thus, the present study does not support introducing carbohydrate loading in this patient group, especially due to the worrying inferior RFS observed in the carbohydrate group.

The strengths of the biological model described above are that it allows changes in the breast tumor to be assessed after manipulating the metabolic environment pre-operatively; thus, it combines the assessment of primary tumor characteristics in concert with systemic metabolic changes. The stable nature of insulin c-peptide also compensated for the more short-lived insulin and IGF. This may explain the more robust nature of insulin c-peptide in the various analyses.

The present study has some weaknesses. First, the number of patients in the intervention arm turned out to be lower than calculated in the power analysis. This may have introduced a type II error in the various statistical analyses. Furthermore, the low number of events and patients at risk in the various survival analyses requires caution in interpreting the results. Moreover, the unbalanced number of participants in the carbohydrate group and fasting group may have introduced confounders. However, as all basic characteristics were evenly distributed between the two study arms, the risk for such confounders is probably quite low. In addition, the proportion of missing data was very low, which contributes to strengthening the study. Regarding tumor markers, a pre-operative biopsy of the tumor would have turned the patients into their own controls. Thus, we could have addressed several questions raised in the discussion, such as the increased PR-negativity in the carbohydrate group. In future studies, pre-operative biopsy must be included to improve the internal validity of the trial.

Finally, the external validity of the present study is limited to luminal breast cancers with T2 tumors. Thus, the present study should be expanded in a multicenter manner, but only in luminal type breast cancers without the PROM quality of life questionnaire. Moreover, a high insulin c-peptide response to a carbohydrate load may predict high risk for relapse. Future research should pursue this clue by adding metabolomic studies to future research on predictive/prognostic circulating biomarkers for systemic relapse in the minutest state possible [65].

## Conclusion

The goal of this study was to investigate the influence of carbohydrates on the biological characteristics of breast cancer. Our working hypothesis was that pre-operative carbohydrate loading affects proliferation and clinical outcome. In the carbohydrate-loading group, the levels of insulin and insulin-c-peptide were increased, whereas those of IGFB3 were decreased. We found that there were more ER+ patients with MAI ≥ 10 among patients who received pre-operative carbohydrate loading than among those who fasted. In addition, the proportion of PR- patients was higher in the carbohydrate group. In ER+ patients with tumors larger than 2 cm (T2), carbohydrate loading seemed to affect clinical outcome with significantly decreased RFS, BCSS, and OS. Only RFS had enough events to enter into a Cox regression model, in which carbohydrate/fasting status and tumor size were the only independent explanatory factors. However, because this study was not powered for survival outcomes, these analyses must be regarded as suggestive. In addition, caution is needed when interpreting the results due to the small sample size and relatively short follow-up. Intriguingly, the decreased expression of PR in the carbohydrate-loaded group suggests the development of endocrine resistance through signaling via membrane-bound receptors, opening up another possibility for the change in clinical outcome than increased proliferation. Taken together, the results of this study indicate that per-oral carbohydrates given pre-operatively may influence both systemic and tumor biology to the benefit of breast cancer cells. Thus, explorative metabolic investigations that focus on identifying novel biomarkers associated with the observed impairment in clinical outcome are warranted.

## Data Availability

The data that support the findings of this study are available from Stavanger Breast Cancer Research Group, but restrictions apply to the availability of these data, which were used under license for the current study and are not publicly available. However, data are available from the authors upon reasonable request and with permission from Stavanger Breast Cancer Research Group.

## Abbreviations

ATP: Adenosine triphosphate
BCSS: Breast cancer-specific survival
BCT: Breast conservative therapy
CI: Confidence interval
CTC: Circulating tumor cell
DCIS: Ductal carcinoma in situ
EGFR: Epidermal growth factor receptor
EMT: Epithelial mesenchymal transition
ER: Estrogen receptor
ERAS: Enhanced recovery after surgery
FNAC: Fine needle aspiration cytology
HER2: Human epithelial growth factor receptor 2
HES: Hematoxylin-eosin saffron staining
HR: Hazard ratio
IGF1: Insulin-like growth factor 1
IGF1R: Insulin-like growth factor 1 receptor
IGF-R: Insulin-like growth factor receptor
IHC: Immunohistochemistry
IR: Insulin receptor
MAI: Mitotic activity index
MRI: Magnetic resonance imaging
NBCG: Norwegian Breast Cancer Group
NSD: Norwegian Center for Research Data
PPH3: Phosphorylated phosphohistone 3
PR: Progesterone receptor
PROM: Patient-reported outcome measure
RFS: Relapse-free survival
SN: Sentinel node
TIL: Tumor-infiltrating lymphocytes
WHO: World Health Organization

## References

[1] World Health Organization: Breast cancer. Available from: http://www.who.int/cancer/prevention/diagnosis-screening/breast-cancer/en/. Accessed 15 Mar 2019.

[2] Veronesi U, Boyle P, Goldhirsch A, Orecchia R, Viale G (2005). Breast cancer. Lancet..

[3] Breast cancer statistics. Available from: https://www.wcrf.org/int/cancer-facts-figures/data-specific-cancers/breast-cancer-statistics. Accessed April 4, 2019.

[4] National Program for Diagnosis, Treatment and Follow-up of Breast Cancer Patients. [In Norwgian]. Avialable from: https://helsedirektoratet.no/retningslinjer. Accessed 12 Apr 2019.

[5] Sorlie T, Perou CM, Tibshirani R, Aas T, Geisler S, Johnsen H, Hastie T, Eisen MB, van de Rijn M, Jeffrey SS (2001). Gene expression patterns of breast carcinomas distinguish tumor subclasses with clinical implications. Proc Natl Acad Sci U S A.

[6] Imyanitov EN (2004). Mechanisms of breast Cancer. Drug Discov Today.

[7] Gross GE, Boldt DH, Osborne CK (1984). Perturbation by insulin of human breast cancer cell cycle kinetics. Cancer Res.

[8] Rose DP, Vona-Davis L (2012). The cellular and molecular mechanisms by which insulin influences breast cancer risk and progression. Endocr Relat Cancer.

[9] Wairagu PM, Phan AN, Kim MK, Han J, Kim HW, Choi JW, Kim KW, Cha SK, Park KH, Jeong Y (2015). Insulin priming effect on estradiol-induced breast cancer metabolism and growth. Cancer Biol Ther.

[10] Voudouri K, Berdiaki A, Tzardi M, Tzanakakis GN, Nikitovic D (2015). Insulin-like growth factor and epidermal growth factor signaling in breast cancer cell growth: focus on endocrine resistant disease. Anal Cell Pathol (Amst).

[11] Park J, Euhus DM, Scherer PE (2011). Paracrine and endocrine effects of adipose tissue on cancer development and progression. Endocr Rev.

[12] Tekade RK, Sun X (2017). The Warburg effect and glucose-derived cancer theranostics. Drug Discov Today.

[13] Bartrons R, Simon-Molas H, Rodriguez-Garcia A, Castano E, Navarro-Sabate A, Manzano A, Martinez-Outschoorn UE (2018). Fructose 2,6-Bisphosphate in Cancer cell metabolism. Front Oncol.

[14] Mallik R, Chowdhury TA (2018). Metformin in cancer. Diabetes Res Clin Pract.

[15] Goodwin PJ, Ennis M, Pritchard KI, Trudeau ME, Koo J, Taylor SK, Hood N (2012). Insulin- and obesity-related variables in early-stage breast cancer: correlations and time course of prognostic associations. J Clin Oncol.

[16] Belfiore A, Frasca F, Pandini G, Sciacca L, Vigneri R (2009). Insulin receptor isoforms and insulin receptor/insulin-like growth factor receptor hybrids in physiology and disease. Endocr Rev.

[17] Klintman M, Strand C, Ahlin C, Beglerbegovic S, Fjallskog ML, Grabau D, Gudlaugsson E, Janssen EA, Lovgren K, Skaland I (2013). The prognostic value of mitotic activity index (MAI), phosphohistone H3 (PPH3), cyclin B1, cyclin a, and Ki67, alone and in combinations, in node-negative premenopausal breast cancer. PLoS One.

[18] Jonsdottir K, Assmus J, Slewa A, Gudlaugsson E, Skaland I, Baak JP, Janssen EA (2014). Prognostic value of gene signatures and proliferation in lymph-node-negative breast cancer. PLoS One.

[19] Awad S, Varadhan KK, Ljungqvist O, Lobo DN (2013). A meta-analysis of randomised controlled trials on preoperative oral carbohydrate treatment in elective surgery. Clin Nutr.

[20] Baum M, Demicheli R, Hrushesky W, Retsky M (2005). Does surgery unfavourably perturb the "natural history" of early breast cancer by accelerating the appearance of distant metastases?. Eur J Cancer.

[21] Smith MD, McCall J, Plank L, Herbison GP, Soop M, Nygren J. Preoperative carbohydrate treatment for enhancing recovery after elective surgery. Cochrane Database Syst Rev. 2014;(8):1–25. 10.1002/14651858.CD009161.pub2 PMC11060647 25121931

[22] Pogatschnik C, Steiger E (2015). Review of preoperative carbohydrate loading. Nutr Clin Pract.

[23] Retsky MW, Demicheli R, Hrushesky WJ, Baum M, Gukas ID (2008). Dormancy and surgery-driven escape from dormancy help explain some clinical features of breast cancer. APMIS..

[24] Cell Biology by the Numbers: How long do the different stages of the cell cycle take? Available from: http://book.bionumbers.org/how-long-do-the-different-stages-of-the-cell-cycle-take/ Accessed 5 Apr 2019.

[25] Spark Notes: The Cell Cycle Topics - Duration of the Cell Cycle. Avaialble from: https://www.sparknotes.com/biology/cellreproduction/cellcycle/section2/ Accessed April 15, 2019.

[26] Lakhani SR, Ellis IO, Scnhitt SJ, Puay HT, van de Vijver MJ (2012). World Health Organization Classification of Tumors. WHO classification of tumors in the breast.

[27] Skaland I, Janssen EA, Gudlaugsson E, Klos J, Kjellevold KH, Soiland H, Baak JP (2009). Validating the prognostic value of proliferation measured by Phosphohistone H3 (PPH3) in invasive lymph node-negative breast cancer patients less than 71 years of age. Breast Cancer Res Treat.

[28] Baak JP, van Diest PJ, Ariens AT, van Beek MW, Bellot SM, Fijnheer J, van Gorp LH, Kwee WS, Los J, Peterse HC (1989). The multicenter morphometric mammary carcinoma project (MMMCP). A nationwide prospective study on reproducibility and prognostic power of routine quantitative assessments in the Netherlands. Pathol Res Pract.

[29] Skaland I, Janssen EA, Gudlaugsson E, Klos J, Kjellevold KH, Soiland H, Baak JP (2007). Phosphohistone H3 expression has much stronger prognostic value than classical prognosticators in invasive lymph node-negative breast cancer patients less than 55 years of age. Mod Pathol.

[30] Bol MG, Baak JP, Rep S, Marx WL, Kruse AJ, Bos SD, Kisman O, Voorhorst FJ (2002). Prognostic value of proliferative activity and nuclear morphometry for progression in TaT1 urothelial cell carcinomas of the urinary bladder. Urology..

[31] Salgado R, Denkert C, Demaria S, Sirtaine N, Klauschen F, Pruneri G, Wienert S, Van den Eynden G, Baehner FL, Penault-Llorca F (2015). The evaluation of tumor-infiltrating lymphocytes (TILs) in breast cancer: recommendations by an international TILs working group 2014. Ann Oncol.

[32] Goldhirsch A, Winer EP, Coates AS, Gelber RD, Piccart-Gebhart M, Thurlimann B, Senn HJ, Panel M (2013). Personalizing the treatment of women with early breast cancer: highlights of the St Gallen international expert consensus on the primary therapy of early breast Cancer 2013. Ann Oncol.

[33] Soiland H, Janssen EA, Korner H, Varhaug JE, Skaland I, Gudlaugsson E, Baak JP, Soreide JA (2009). Apolipoprotein D predicts adverse outcome in women > or=70 years with operable breast cancer. Breast Cancer Res Treat.

[34] Janssen EA, van Diest PJ, Soiland H, Gudlaugson E, Nysted A, Voorhorst FJ, Vermorken JB, Soreide JA, Baak JP (2006). Success predictors of adjuvant chemotherapy in node-negative breast cancer patients under 55 years. Cell Oncol.

[35] Warburg O, Posener K, Negelein E (1924). Ueber den stoffwechhsel der tumoren. Biochem Z.

[36] The Nobel Prize. The Nobel Prize in Physiology or Medicine 1931. Available from: https://www.nobelprize.org/prizes/medicine/1931/summary/. Accessed 30 Mar 2019.

[37] Skaland I, Janssen EA, Gudlaugsson E, Hui Ru Guo L, Baak JP (2009). The prognostic value of the proliferation marker phosphohistone H3 (PPH3) in luminal, basal-like and triple negative phenotype invasive lymph node-negative breast cancer. Cell Oncol.

[38] Balkenhol Maschenka C. A., Bult Peter, Tellez David, Vreuls Willem, Clahsen Pieter C., Ciompi Francesco, van der Laak Jeroen A. W. M. (2019). Deep learning and manual assessment show that the absolute mitotic count does not contain prognostic information in triple negative breast cancer. Cellular Oncology.

[39] Huang J, Morehouse C, Streicher K, Higgs BW, Gao J, Czapiga M, Boutrin A, Zhu W, Brohawn P, Chang Y (2011). Altered expression of insulin receptor isoforms in breast cancer. PLoS One.

[40] Marzec KA, Baxter RC, Martin JL (2015). Targeting insulin-like growth factor binding Protein-3 signaling in triple-negative breast Cancer. Biomed Res Int.

[41] Law JH, Habibi G, Hu K, Masoudi H, Wang MY, Stratford AL, Park E, Gee JM, Finlay P, Jones HE (2008). Phosphorylated insulin-like growth factor-i/insulin receptor is present in all breast cancer subtypes and is related to poor survival. Cancer Res.

[42] Hanahan D, Weinberg RA (2011). Hallmarks of cancer: the next generation. Cell..

[43] Tan EJ, Olsson AK, Moustakas A (2015). Reprogramming during epithelial to mesenchymal transition under the control of TGFbeta. Cell Adhes Migr.

[44] Lumachi F, Ermani M, Brandes AA, Basso S, Basso U, Boccagni P (2001). Predictive value of different prognostic factors in breast cancer recurrences: multivariate analysis using a logistic regression model. Anticancer Res.

[45] Selli C, Turnbull AK, Pearce DA, Li A, Fernando A, Wills J, Renshaw L, Thomas JS, Dixon JM, Sims AH (2019). Molecular changes during extended neoadjuvant letrozole treatment of breast cancer: distinguishing acquired resistance from dormant tumours. Breast Cancer Res.

[46] Zielinska HA, Bahl A, Holly JM, Perks CM (2015). Epithelial-to-mesenchymal transition in breast cancer: a role for insulin-like growth factor I and insulin-like growth factor-binding protein 3?. Breast Cancer (Dove Med Press).

[47] Sorokin AV, Chen J (2013). MEMO1, a new IRS1-interacting protein, induces epithelial-mesenchymal transition in mammary epithelial cells. Oncogene..

[48] Kim HJ, Litzenburger BC, Cui X, Delgado DA, Grabiner BC, Lin X, Lewis MT, Gottardis MM, Wong TW, Attar RM (2007). Constitutively active type I insulin-like growth factor receptor causes transformation and xenograft growth of immortalized mammary epithelial cells and is accompanied by an epithelial-to-mesenchymal transition mediated by NF-kappaB and snail. Mol Cell Biol.

[49] Liu Y, Dhall S, Castro A, Chan A, Alamat R, Martins-Green M (2018). Insulin regulates multiple signaling pathways leading to monocyte/macrophage chemotaxis into the wound tissue. Biol Open.

[50] Papavasiliou P, Fisher T, Kuhn J, Nemunaitis J, Lamont J (2010). Circulating tumor cells in patients undergoing surgery for hepatic metastases from colorectal cancer. Proc (Baylor Univ Med Cent).

[51] Iida Masafumi, Tsuboi Kouki, Niwa Toshifumi, Ishida Takanori, Hayashi Shin-ichi (2018). Compensatory role of insulin-like growth factor 1 receptor in estrogen receptor signaling pathway and possible therapeutic target for hormone therapy-resistant breast cancer. Breast Cancer.

[52] Cui X, Schiff R, Arpino G, Osborne CK, Lee AV (2005). Biology of progesterone receptor loss in breast cancer and its implications for endocrine therapy. J Clin Oncol.

[53] Montgomery M. Srinivasan A. Epigenetic Gene Regulation by Dietary Compounds in Cancer Prevention. Adv Nutr. 2019. E-pub May 17, 2019. 10.1093/advances/nmz046. 10.1093/advances/nmz046 PMC6855955 31100104

[54] Giuliano M, Schifp R, Osborne CK, Trivedi MV (2011). Biological mechanisms and clinical implications of endocrine resistance in breast cancer. Breast.

[55] Ok JH, Lee H, Chung HY, Lee SH, Choi EJ, Kang CM, Lee SM (2018). The potential use of a ketogenic diet in pancreatobiliary cancer patients after pancreatectomy. Anticancer Res.

[56] Sun W, Lu J, Wu S, Bi Y, Mu Y, Zhao J, Liu C, Chen L, Shi L, Li Q (2016). Association of insulin resistance with breast, ovarian, endometrial and cervical cancers in non-diabetic women. Am J Cancer Res.

[57] AlFakeeh A, Brezden-Masley C (2018). Overcoming endocrine resistance in hormone receptor-positive breast cancer. Curr Oncol.

[58] Alimova IN, Liu B, Fan Z, Edgerton SM, Dillon T, Lind SE, Thor AD (2009). Metformin inhibits breast cancer cell growth, colony formation and induces cell cycle arrest in vitro. Cell Cycle.

[59] Sharma A, Bandyopadhayaya S, Chowdhury K, Sharma T, Maheshwari R, Das A, Chakrabarti G, Kumar V, Mandal CC (2019). Metformin exhibited anticancer activity by lowering cellular cholesterol content in breast cancer cells. PLoS One.

[60] Yam C, Esteva FJ, Patel MM, Raghavendra AS, Ueno NT, Moulder SL, Hess KR, Shroff GS, Hodge S, Koenig KH (2019). Efficacy and safety of the combination of metformin, everolimus and exemestane in overweight and obese postmenopausal patients with metastatic, hormone receptor-positive, HER2-negative breast cancer: a phase II study. Investig New Drugs.

[61] Scherbakov AM, Sorokin DV, Tatarskiy VV, Prokhorov NS, Semina SE, Berstein LM, Krasil'nikov MA (2016). The phenomenon of acquired resistance to metformin in breast cancer cells: the interaction of growth pathways and estrogen receptor signaling. IUBMB Life.

[62] Hundshammer C, Braeuer M, Muller CA, Hansen AE, Schillmaier M, Duwel S, Feuerecker B, Glaser SJ, Haase A, Weichert W (2018). Simultaneous characterization of tumor cellularity and the Warburg effect with PET, MRI and hyperpolarized (13)C-MRSI. Theranostics..

[63] Fujii T, Yanai K, Tokuda S, Nakazawa Y, Kurozumi S, Obayashi S, Yajima R, Hirakata T, Shirabe K (2018). Relationship between FDG uptake and neutrophil/lymphocyte ratio in patients with invasive ductal breast Cancer. Anticancer Res.

[64] Norwegian Health Directorate. Trajectory for the Treatment of Breast Cancer in Norway. [In Norwegian]. Available from: https://helsedirektoratet.no/retningslinjer/pakkeforlop-for-brystkreft. Accessed 12 Mar 2019. 2015.

[65] Lunde S, Helland T, Jonassen J, Haugstøyl M, Austdal M, Lode K, Hagen KB, Gripsrud BH, Lind RA, Gjerde J (2018). A prospective, longitudinal, breast cancer biobank (PBCB) in western Norway.

